# Using virtual reality to augment perception, enhance sensorimotor adaptation, and change our minds

**DOI:** 10.3389/fnsys.2014.00056

**Published:** 2014-04-08

**Authors:** W. Geoffrey Wright

**Affiliations:** Physical Therapy and Bioengineering, Motion Analysis and Perception Laboratory, Temple UniversityPhiladelphia, PA, USA

**Keywords:** virtual reality environment, vection, sensorimotor integration, motor adaptation, prism adaptation cross-modal processing, posture control, brain augmentation

## Abstract

Technological advances that involve human sensorimotor processes can have both intended and unintended effects on the central nervous system (CNS). This mini review focuses on the use of virtual environments (VE) to augment brain functions by enhancing perception, eliciting automatic motor behavior, and inducing sensorimotor adaptation. VE technology is becoming increasingly prevalent in medical rehabilitation, training simulators, gaming, and entertainment. Although these VE applications have often been shown to optimize outcomes, whether it be to speed recovery, reduce training time, or enhance immersion and enjoyment, there are inherent drawbacks to environments that can potentially change sensorimotor calibration. Across numerous VE studies over the years, we have investigated the effects of combining visual and physical motion on perception, motor control, and adaptation. Recent results from our research involving exposure to dynamic passive motion within a visually-depicted VE reveal that short-term exposure to augmented sensorimotor discordance can result in systematic aftereffects that last beyond the exposure period. Whether these adaptations are advantageous or not, remains to be seen. Benefits as well as risks of using VE-driven sensorimotor stimulation to enhance brain processes will be discussed.

## Introduction

The use of technology to augment brain function has a nebulous history, in part, because the term *brain augmentation* can be defined in so many ways. It implies an enhancement of brain function, but how it is measured introduces some uncertainty. To clarify this further, it should be recognized that the function of the brain is nothing short of control of the entire body and all one’s experiences, both internal processes and externally-directed actions. Therefore augmenting brain function could include a wide range of easy or difficult to identify enhancements, such as boosting immune system response, improving mood, memory, or perception, optimizing motor control, or increasing sub-optimal function after injury. To make brain augmentation even harder to define, sometimes the only measure we have of its effective implementation is through observation of external behavior.

A simple definition of brain augmentation could be enhancement of sensation, such as the use of spectacles to improve myopia. Most glasses wearers will be familiar with the initial adaptation to a slight magnification and/or shift in close-up visual space that occurs with new glasses. This type of adaptation is even more evident when using light-refracting prism glasses to intentionally shift the visually perceived world. Facilitation of adaptive processes could be considered another form of brain augmentation. For example, prism adaptation has been used to correct strabismus by helping the eyes accomplish perceptual convergence and reducing the angle of squint (Pigassou, 1972). In hemispatial neglect wearing prism glasses can augment the brain’s ability to adapt its sensorimotor representation of the external world and reduce the perceptual deficit (Rossetti et al., 1998).

Augmenting brain function by enhancing perception or compensating for a deficit is an area in which virtual reality (VR) technology is also being applied. Similar to prism adaptation, VR technology can be used to alter the egocentric and allocentric representations of the world (Castiello et al., 2004). In this paper, the issue of identifying enhancement in the realm of sensory perception, motor control, and sensorimotor adaptation by using virtual environments (VE) will be reviewed. The sensorimotor and perceptual processes that may cause these changes will be examined while also highlighting the difficulty that goes along with defining, measuring, and quantifying enhancement. Not all changes are enhancement, since some are not adaptive or are accompanied by unwanted degradation of other functions or cause unintended responses.

## Augmenting perception

A fundamental aspect of creating an immersive experience within a VE is in creating a sense of three-dimensionality so that one believes one can move about within the virtual world. A common barrier to this is that one may wish to travel further in the VE than is possible in a limited space (e.g., a flight simulation). Although the perception of self-motion typically occurs as a result of combined visual and physical motion stimuli, its long been known that visually-induced self-motion perception (i.e., vection) can occur in the absence of actual motion (Mach, 1875; Young et al., 1973; Pavard and Berthoz, 1977; Howard, 1986; Frigon and Delorme, 1992; Harris et al., 2000; Palmisano, 2002; Wright, 2002). However, optic flow when walking about the real world is different than when rotating the eyes or head while the body remains stationary (Gibson, 1954). That our central nervous system (CNS) can accurately detect this difference likely evolved as an essential part of navigating through the world searching for food or mates and avoiding danger. The exact selective pressures that shaped these neural processes can only be inferred, however, its very likely that in the pre-modern world earth-bound species (flightless and terrestrial) were less frequently exposed to the visual-vestibular discordance that occurs during passive motion to which we are often exposed in the modern world (e.g., during any vehicle transport). Although the multiple sensory inputs that occur during self-motion do not always have a prescribed relation, they co-occur with greater probability in certain relations to one another than others. For example, terrestrial animals experience sustained vection (very low frequency) in a direction orthogonal to vertical more frequently than parallel to it. Furthermore, the sensory organs are oriented upright relative to gravity more frequently than inverted. Despite this, it seems there was a strong selective advantage for omnidirectional sensory organs that could be mapped to each other rather flexibly. One need only don a pair of prism glasses to experience visuomotor remapping that makes even a completely inverted world eventually feel “normal” (Stratton, 1896). This sensorimotor lability can be used to induce a compelling sense of immersion in a VE even when sensory inputs are incongruent, sub-threshold, or absent.

The flexible, adaptable sensorimotor integration described above is how the brain’s perceptual abilities can be augmented in a VE. For example, self-motion perception can be induced even when sensory input is below threshold. In a VE, the inertial stimulus is often sub-threshold or even absent, yet perception of self-motion is still experienced. In our VE studies, we have tested how this perception is affected by manipulating the concordance of visual and inertial sensory inputs (see Table 1; Wright, 2002, 2009; Wright and Glasauer, 2003, 2006; Wright and Schneider, 2009). To enhance the level of immersion, subjects viewed a high-fidelity, realistic visual scene from a first person perspective while wearing a head-mounted display (HMD; Wright et al., 2005, 2006, 2009). To further enhance the perceptual experience, we drew on previously established knowledge about motion perception. The visual system primarily transduces velocity information from the visual stimulus, and is less sensitive to acceleration (Berthoz et al., 1975; Telford et al., 1992; Warren and Kurtz, 1992). Acceleration information is primarily derived from the inertial stimulus, which even in the absence of vision plays an important part of the experience of motion (Wright et al., 2005). Gravitoinertial input resulting from physical motion and background gravity stimulate the vestibular and somatosensory systems (i.e., the graviceptors) which provide linear acceleration and rotation information (Guedry, 1974). But like the visual system, there is an overlap in what qualifies as an adequate stimulus, as suggested by the fact that graviceptors may also be sensitive to velocity cues (Jones and Young, 1978). Our investigations suggest that combinations of physical and visual stimulation can result in sensory summation. This can increase how compelling perceived self-motion is when stimuli are matched directionally and temporally (Wright et al., 2005). However, we have also found that perceived self-motion can be enhanced by adding together discordant stimuli, such as by combining physical tilt with visually-depicted translation (Wright et al., 2009). In fact, the “compellingness” of visually-induced self-motion can be enhanced in the direction of the visual stimulus by increasing the amplitude of a discordant inertial input. This is true even if the visual and inertial vectors are 90–180° out of alignment with each other (Wright et al., 2005; Wright, 2009; Ash et al., 2011).

**Table 1:** **Perceptual and/or motor behavior in immersive VE**.

	**Study**	**Visual Stimulus**	**Physical Stimulus**	**Instructed response (DV)**	**Unintended Response**
Wright and Glasauer (2003)	Static or Roll tilting VE scene	Roll tilt of whole body while sitting	Subjective vertical (joystick/glass orientation)	Motor response depends on type of object wielded even if similar size and weight
Wright et al. (2005)	Up-down translation of VE scene depicting various amplitudes of motion	Up-down whole body translation either matching or mismatching visual amplitude	Subjective estimate of self-motion amplitude	Visual-vestibular weighting not always linear summation
Wright et al. (2009)	Left-right translation of VE scene	Roll tilt of whole body while sitting	Subjective vertical (joystick orientation)	Joystick Translations
Wright and Schneider (2009)	Left-right or up-down translation of VE scene	None	Subjective vertical (joystick orientation)	Joystick Translations
Wright (2009)	Left-right translation of VE scene	Up-down whole body translation of various amplitudes	Subjective estimate of self-motion amplitude	Left-right self-motion perception increased as physical up-down motion increased
Wright et al. (2013)	Left-right translation of VE scene	Fore-aft whole body translation while sitting	Stabilize head (yaw, pitch, roll angular velocity)	Yaw head movement
Wright (2013)	Fore-aft translation of VE scene in 3-walled, earth-fixed cave	Left-right whole-body translation while standing	Maintain balance (center of pressure)	Postural response to visual+physical stimulus. Postural aftereffects during post-adaptation visual stimulation

Another contributing factor that augments the VE-user’s immersion by enhancing the self-motion experience is predicated on the idea of tilt-translation ambiguity (Young et al., 1984; Parker et al., 1985; Merfeld et al., 2005; Holly et al., 2006). Accurate perception of tilt versus translation is obfuscated in part because gravitational and inertial accelerations are physically indistinguishable, and in part because the adequate stimulus for the visual system is velocity-dependent while the adequate stimulus for graviceptors is acceleration-dependent. Thus, tilt in a gravitational field could conceivably be perceived as translation. However, when multiple sensory inputs are combined, a unified sense of self-motion can be derived. Whether this perception is rotation or translation or some combination of both is, in part, determined by spatial, temporal, and cognitive factors (Wertheim et al., 2001; Wright et al., 2006; Holly and McCollum, 2008; Riecke, 2009). The perception also depends on the independence of the velocity and acceleration vectors. In nature, a positive velocity could be experienced with an infinite number of positive or negative acceleration vectors. This is important because the visual system’s velocity-sensitive and the vestibular system’s acceleration-sensitive. All these factors contribute to how VE can be used to induce unusual self-motion perceptions, despite the fact that the sensory pattern has never been experienced (Wright, 2009). This fact has been capitalized on by IMAX®-based rides for decades, where an individual can experience compelling self-motion while completely stationary; moreover, the perception can be further enhanced when subtle movements of the theater seat are added.

The phenomenological evidence above suggests that veridical self-motion perception is dependent on the integration of multiple sensory inputs that do not have a prescribed relation to one another. Moreover, perception can be extended beyond the normal limits of the senses by combining sensory inputs in VE to induce non-veridical self-motion perception. A sense of immersion in a VE can be enhanced by the belief that movement within the VE is actually happening. But if the VE is augmenting perception of a “false” reality, does this also alter one’s actions? And more importantly, is there a real-world benefit? The first question has been brought up before (Stanney et al., 1998; Cohn et al., 2000) but lately the focus of attention has shifted to the latter question. Many researchers and clinicians have embarked upon systematic investigation of how VR can augment the brain’s ability to recover from disease and injury (e.g., Liebermann et al., 2012). In the next sections we delve into these questions, first by determining whether motor behaviors are altered by VE immersion then by looking at sensorimotor adaptation within a VE.

## The perception-action link in VE

Perception can be defined as the organization of sensory stimuli (both externally received or internally generated) resulting in a conscious awareness of a phenomenon. Perception and action are tightly linked processes, with the interaction of sensory input and motor output being fundamental to the calibration of fine and gross motor skills. Accurate perception of the body’s movements is critical to fine-tuning motor skills. However, there are many examples of dissociations between perception and action (e.g., Goodale and Milner, 1992; Merfeld et al., 2005). Thus, even though perception of self-motion and orientation can be altered in a VE, how it affects motor output has required investigation. In a series of studies, we looked at manual motor control, head stabilization, and postural control while immersed in a VE to evaluate whether the perception-action link reliably occurs in VE.

Much like in the perceptual studies, we tested conditions that combined visually-depicted motion both with and without actual physical motion. Subjects performed a manual motor control task while sitting in a tiltable motion device and viewing a visual depiction of linear translation in an HMD (Wright and Glasauer, 2003, 2006; Wright et al., 2009; Wright and Schneider, 2009). During this VE immersion, subjects were instructed to align a handheld object to the perceived vertical. Perceptual reports confirmed self-translation to be more compelling when physical tilt of the subject was added to the linear visual motion (Wright et al., 2009; Wright and Schneider, 2009). But interestingly, despite being asked only to align the handheld object to vertical, subjects also showed automatic, unconscious translation of the unconstrained arm. These unintended motor actions were entrained with the visually-depicted motion, as if experiencing accelerations due to actual translation (see Table 1). Although its been shown that constant velocity visual rotation in a VE can affect the direction of reaching (Cohn et al., 2000; Dvorkin et al., 2009), our findings also revealed that vision could induce vection with both velocity and acceleration components. Automatic motor responses occurred as if a compensatory force was required to counter the illusory acceleration. Moreover, the more compelling the perceived self-motion, the larger the motor response.

We were also interested in determining how VE immersion might affect other motor control processes such as head stabilization (Wright et al., 2013) and whole-body postural control (Wright, 2013). In one study, subjects were exposed to physical accelerations while viewing a directionally discordant visual scene. Significant head stabilization responses were dependent on the direction of visual flow even though visual input was discordant with the physical stimulus (Wright et al., 2013). In another study applying a similar experimental test protocol to investigate posture control, we paired visually-depicted translation in one direction with an orthogonally directed translation of the support surface. This cross-axis stimulation resulted in a postural response in standing humans that showed entrainment with both the visual and the support surface stimuli (Wright, 2013). These findings may not be unexpected, since studies pre-dating VR technology have shown that postural control can be entrained to a visual stimulus by simulating movement of the entire room, when the floor is kept stationary (Lishman and Lee, 1973). Furthermore, the postural response can be influenced by the characteristics of the discordant visual input (Lestienne et al., 1977). If an immersive VE is used the postural response to a visual input can be potentiated even if the input is temporally discordant with surface movement (Keshner et al., 2004). Together these results suggest that VE can be used to induce a motor response to a non-veridical virtual input, even in the presence of a strong destabilizing physical stimulus that requires an appropriate postural response to keep from falling. Thus, VE can cause an unintended postural response leading to instability.

## Inducing sensorimotor adaptation and maladaptive aftereffects

To study postural adaptation in VE, we exposed subjects to cross-axis postural stimulation for an extended period of time. By looking for aftereffects, as is often done in prism-adaptation research, we wished to see if the postural response seen during discordant stimulation involved adaption or was simply a transient response that would disappear as soon as one of the stimuli was removed (Wright, 2013). The plasticity of the CNS, which allows discordance to be reduced by sensorimotor re-mapping, is present at birth and plays an essential role in shaping motor development through interaction with the environment (Held and Freedman, 1963). Seminal work on visuospatial adaptation using light-refracting prism glasses dating back a century and a half (von Helmholtz, 1867; Stratton, 1896) has shown us that although neural plasticity may diminish with age, this form of plasticity does not have a critical period; rather to some extent it lasts well into adulthood (Hardt et al., 1971). Therefore, determining whether VE exposure will automatically cause sensorimotor adaptation is important. If one considers VE usage in everyday life, for training purposes, or for rehabilitation, one might expect very large exposure times, which could have long lasting aftereffects.

We exposed standing subjects for an extended period of time (5 min) to the cross-axis stimulation. After the adaptation period, we stopped the dynamic support stimulation while subjects continued to view the same visual translation stimulus as before. What was found in the post-adaptation phase is that subjects indeed showed an aftereffect in the COP, which was rotated as much as 45° from the direction of the visual stimulation. This finding is complementary to the finding which showed sensorimotor adaptation of the vestibulo-ocular reflex (VOR) in monkeys after prolonged pairing of physical rotation with an orthogonally directed optic flow (Wei and Angelaki, 2001). The evidence from our study on humans show that long lasting (>2 min) whole-body sensorimotor adaption can also be induced after prolonged exposure (<5 min) to discordant combinations of inertial and visual stimulation in a VE (Wright, 2013). Preliminary evidence suggests that these adaptations may even last for a few days. Specifically, after a few exposures staggered over multiple days, aftereffects appear immediately upon re-immersion into the VE without requiring a fresh dose of cross-axis adaptation.

This raises the question whether these residual aftereffects seen in healthy individuals who spend extended time in a VE are adaptive in the real world or unwanted aftereffects. Inaccurate automatic motor responses due to recalibration of visual, vestibular, and somatosensory coordination certainly involves a risk. However, for those with a perceptual or motor deficit, VE immersion may move them from a maladaptive state towards an adaptive one. Although this requires further investigation, what is apparent is that VE can be used to facilitate the brain’s natural ability to adapt to changes in the sensory environment. In fact, VE-based brain augmentation applications have already begun to appear in clinical applications. They have been used to ameliorate hemi-neglect symptoms by remapping the neglected space (Castiello et al., 2004), thus reducing a perceptual deficit, and improving function.

## Conclusions

The fidelity of immersive VE has increased with amazing speed over the last two decades. One need only play the latest release of any popular first person shooter or sports-themed video game to be astounded by its verisimilitude. Because of the level of immersion one can experience, the question arises as to how quickly our CNS adapts to such simulated reality. More importantly, how robust are these adaptations, not only to the accurate attributes of the manufactured environments, but also to their inaccuracies. Even if visual fidelity such as spatial resolution, update rates, perspective geometry, monocular and binocular cues, et cetera are all perfectly programmed to match reality in a VE, the absence of inertial cues to accompany simulated translation through such environments will affect how the CNS calibrates the perceptuomotor system to these non-veridicalities. In other words, exposure to a VE will automatically cause sensorimotor adaptation, whether desired or not. But hopefully, VE’s ability to augment brain functions by enhancing perception, eliciting automatic motor behavior, and inducing sensorimotor adaptation can be applied in useful ways.

Trusting what we can measure as evidence of brain enhancement may not solve the problem of defining brain augmentation, when short-term benefits may accompany long-term deficits. Conversely, long-term enhancements may be overlooked because of a more immediate decrement in brain function that can happen as the brain adapts to or learns a new technology. What can be certain is that benefits from brain augmentation, if understood and applied, can be far-reaching. If used to recover loss of function following neurological damage, brain augmentation can potentially bring function back to healthy levels. The ways in which scientist, technologist, and clinicians are applying VR technology to rehabilitation is already happening and the hopes are that the individuals involved in this collective creative process will have the foresight to exploit the potential of neural plasticity, while recognizing that not all change is good.

## Conflict of interest statement

The author declares that the research was conducted in the absence of any commercial or financial relationships that could be construed as a potential conflict of interest.
